# Postnatal liver growth and regeneration are independent of *c-myc *in a mouse model of conditional hepatic *c-myc *deletion

**DOI:** 10.1186/1472-6793-12-1

**Published:** 2012-03-07

**Authors:** Jennifer A Sanders, Christoph Schorl, Ajay Patel, John M Sedivy, Philip A Gruppuso

**Affiliations:** 1 Department of Pediatrics, Rhode Island Hospital and Brown University, Providence, RI 02903, USA; 2 Department of Molecular Biology, Cell Biology and Biochemistry, Brown University, Providence, RI 02912, USA; 3 Center for Genomics and Proteomics, Brown University, Providence, RI 02912, USA; 4 Department of Pathology, MacNeal Hospital, Berwyn, IL, USA

## Abstract

**Background:**

The transcription factor *c-myc *regulates genes involved in hepatocyte growth, proliferation, metabolism, and differentiation. It has also been assigned roles in liver development and regeneration. In previous studies, we made the unexpected observation that c-Myc protein levels were similar in proliferating fetal liver and quiescent adult liver with c-Myc displaying nucleolar localization in the latter. In order to investigate the functional role of c-Myc in adult liver, we have developed a hepatocyte-specific *c-myc *knockout mouse, *c-myc^fl/fl^*;*Alb*-*Cre*.

**Results:**

Liver weight to body weight ratios were similar in control and *c-myc *deficient mice. Liver architecture was unaffected. Conditional *c-myc *deletion did not result in compensatory induction of other *myc *family members or in c-Myc's binding partner Max. Floxed *c-myc *did have a negative effect on *Alb*-Cre expression at 4 weeks of age. To explore this relationship further, we used the Rosa26 reporter line to assay Cre activity in the *c-myc *floxed mice. No significant difference in Alb-Cre activity was found between control and *c-myc^fl/fl ^*mice. c*-myc *deficient mice were studied in a nonproliferative model of liver growth, fasting for 48 hr followed by a 24 hr refeeding period. Fasting resulted in a decrease in liver mass and liver protein, both of which recovered upon 24 h of refeeding in the c*-myc^fl/fl^;Alb*-Cre animals. There was also no effect of reducing *c-myc *on recovery of liver mass following 2/3 partial hepatectomy.

**Conclusions:**

c-Myc appears to be dispensable for normal liver growth during the postnatal period, restoration of liver mass following partial hepatectomy and recovery from fasting.

## Background

The Myc family includes three closely related genes, *c-myc, L-myc*, and *N-myc*, which have been shown to have similar biological activities. The three Myc proteins are basic helix-loop-helix leucine zipper transcription factors that heterodimerize with a binding partner, Max, to bind DNA and either activate or repress the transcription of a large set of target genes [1-3]. An additional member of the family, *B-myc*, encodes a protein that is homologous to the N-terminal domain of c-Myc, but its function remains largely unknown [4]. c-Myc has been shown to regulate genes involved in ribosomal biogenesis, protein translation and the transition from the G0/G1 to S-phase of the cell cycle suggesting that c-Myc has a functional role in the coordination of cellular growth and proliferation. The expression of *c-myc *is, in general, tightly regulated. Proliferating cells contain high levels of this protein, while the level of c-Myc is significantly decreased as cells growth arrest and differentiate [3]. Dysregulated expression of *c-myc *is associated with the development of many tumors in rodents and humans, including hepatocellular carcinoma [5-7].

c-Myc has been implicated as a regulator of hepatocyte proliferation, growth and metabolism [8,9]. During the process of liver regeneration, quiescent hepatocytes synchronously enter the cell cycle and undergo one, two or more rounds of replication to restore liver mass [10]. Considered an immediate early gene, *c-myc *expression is induced within 30 minutes following partial hepatectomy and has been suggested to be a key factor in the transcriptional response leading to the progression of hepatocytes from G0/G1 to S phase [11]. Transient overexpression of c-Myc in mouse liver results in hepatocyte enlargement and induction of ribosomal and nucleolar genes [12]. Other studies involving *c-myc *transgenic mice have shown that overexpression of c-Myc in the liver induces hepatic glucose uptake and utilization and can inhibit gluconeogenesis [13,14]. While these studies support a role for c-Myc in hepatocyte growth, ribosomal biogenesis and metabolism, they do not address whether c-Myc is required or whether the effects on these processes were due to superphysiological levels of c-Myc.

Previous studies from our laboratory on the regulation of c-Myc during rat liver development revealed several novel findings. First, in contrast to many other organ systems and cell types, rapidly proliferating fetal and quiescent adult liver contained similar levels of c-Myc protein. In adult hepatocytes, c-Myc was localized to the nucleolus, while fetal hepatocytes displayed diffuse nuclear localization. In addition, c-Myc translocated out of the nucleolus in response to a partial hepatectomy [15,16]. These data led us to hypothesize that hepatic c-Myc may play a functional role in liver other than its well established role in hepatocyte proliferation. In order to examine the function of c-Myc in adult liver, we mated mice in which the *c-myc *locus was floxed to mice expressing Cre recombinase under the control of the albumin promoter. The present paper describes the characterization of these mice.

## Methods

### Animals

*c-myc^fl/fl ^*mice were gifts of I. Moreno de Alboran [17]. *Alb*-Cre and ROSA26 mice were obtained from Jackson Laboratories (Bar Harbor, ME [18,19]). All mice were C57BL/6 strain housed in a pathogen-free facility and maintained on a 12 hr light/dark cycle. *c-myc^fl/fl ^*mice were mated to *Alb*-Cre mice to achieve mice that carried a floxed *c-myc *allele and *Alb*-Cre. Littermates were bred to obtain *c-myc^fl/fl^;Alb*-Cre^+ ^mice and the control mice (*c-myc^fl/fl^;Alb*-Cre^-/-^, *c-myc^+/+^;Alb*-Cre^+^). Progeny were mated to obtain *c-myc^fl/fl^;Alb*-Cre^+/+ ^mice and the control *c-myc^+/+^;Alb*-Cre^+/+ ^mice. Breeding pairs that produced litters consisting exclusively of Cre^+ ^pups for at least 5 successive matings were considered Cre^+/+^. *c-myc^fl/fl^;Alb*-Cre^+/+ ^and the control *c-myc^+/+^;Alb*-Cre^+/+ ^lines were established from these pairs. Pups from both sexes were used for all analyses except 2/3 partial hepatectomy. Blood glucose concentrations were determined using a YSI 2300 STAT plus glucose and lactate analyzer (YSI Life Sciences; Yellow Springs, OH).

For fasting and refeeding experiments, eight week old male and female *c-myc^fl/fl ^*and *c-myc^+/+^;Alb*-Cre expressing mice were fed standard rodent chow *ad libitum *(control) or fasted for 48 hr. Where noted, standard rodent chow was replaced in the cage covers and animals were allowed to feed *ad libitum *for 24 hr (refed). Water was freely available to all mice. Eight to ten week old male *c-myc^fl/fl ^*and *c-myc^+/+ ^Alb*-Cre expressing mice were anesthetized using isoflurane and subjected to 2/3 partial hepatectomy as described by Higgins and Anderson [20]. All mice were killed by exsanguination under isoflurane anesthesia. Carcass and liver weights were recorded. The liver was divided and fixed in 10% neutral buffered formalin or flash frozen in liquid nitrogen before being stored at -70°C.

To assess Cre activity, female *c-myc^fl/fl^;Alb*-Cre^+/+ ^and *c-myc^+/+^;Alb*-Cre^+/+ ^were crossed with male ROSA26 mice. Mice were further mated to obtain the following genotypes used for study, *c-myc^fl/fl^;Alb*-Cre^*+*^;ROSA26*^+ ^*and *c-myc^+/+^;Alb*-Cre^*+*^;ROSA26^*+*^. Mice were genotyped using PCR analysis of tail genomic DNA according to published protocols for *c-myc *[21], *Alb*-Cre transgene [22] and the ROSA26 alleles http://jaxmice.jax.org/protocolsdb/f?p=116:1:3259541732707236::NO:::.

All experiments on mice were performed in accordance with the guidelines of the National Institutes of Health and the Rhode Island Hospital Institutional Animal Care and Use Committee.

### DNA Isolation and qPCR

DNA was isolated from triplicate frozen livers obtained from *c-myc^fl/fl^;Alb*-Cre^-/-^, *c-myc^fl/fl^;Alb*-Cre^± ^and *c-myc^fl/fl^;Alb*-Cre^+/+ ^mice at 4, 8, and 10 weeks of age using the DNeasy kit (Qiagen; Valencia, CA). qPCR reactions were performed in triplicate using 25 ng of DNA, 23 μl SYBR green reaction mix, and the 7500 Real-Time PCR system (Applied Biosystems; Foster City, CA). In order to detect the deletion of the *c-myc^fl ^*allele, primers were designed upstream of the 5' lox P site (primers X and Y) and on either side of the 3' lox P site (NB). The primer sequences are as follows: primer X, 5'-CCTCGCGCCCCTGAA-3'; primer Y, 5'-AACCGCTCAGATCACGACTCA-3'; primer N, 5'-TCCAAACCAGAAACTGAAACATGT-3'; primer B, 5'-ACAATGGGGTCATTTAGGAC-3'. The relative abundance of the *c-myc^fl ^*allele was calculated by the comparative C_T _method using the product generated by primers X and Y as the reference. *c-myc *deletion during liver regeneration was assessed by calculating the ddCt (dCt in liver removed at the time of hepatectomy-- minus the dCt in regenerating liver from the same animal) for triplicate mice.

### RNase protection assay

Total RNA was isolated from triplicate frozen livers obtained from 8 and 10 week old *c-myc^+/+ ^*and *c-myc^fl/fl ^Alb*-Cre expressing mice as previously described [15]. RNase protection assays were performed using the mMyc multiprobe template with yeast tRNA as a negative control (BD Biosciences; San Diego, CA). L32 was used as an internal control to normalize expression data. Quantification of bands was performed by digital analysis using LabWorks software (UVP; Upland, CA).

### RT-qPCR

Total RNA was isolated from frozen livers obtained from 4, 8, and 10 week old control (*c-myc^fl/fl^;Alb*-Cre^-/- ^and *c-myc^+/+^;Alb*-Cre) and *c-myc^fl/fl^;Alb*-Cre mice using the RiboPure Kit (Ambion; Foster City, CA). RNA was cleaned using the RNeasy kit (Qiagen) and cDNA synthesized using random hexamers and the TaqMan Reverse Transcription kit (Applied Biosystems). Primer sequences used for amplification of *cre *were 5'-CGATGCAACGAGTGATGAGG-3' for the sense primer and 5'-GGCAAACGGACAGAAGCATT-3' for the antisense primer. Mouse *c-myc *primers were obtained from SABiosciences (Frederick, MD). The internal standard *GAPDH *was amplified using the following primer sequences, 5'-TCCAGTATGACTCCACTCACGG-3' and 5'-TCGCTCCTGGAAGATGGTG-3'. The relative abundance of *cre *and *c-myc *was calculated by the comparative CT method using *GAPDH *as the reference.

### Histology and image analysis

Liver was fixed in 10% neutral buffered formalin, paraffin embedded, and stained with hemotoxylin and eosin. Immunohistochemistry was performed for Ki-67 using the indirect immuneperoxidase technique. Briefly, sections were deparaffinized and microwaved in 100 mM sodium citrate buffer, pH 6.0 for 20 minutes, followed by incubation in anti-Ki-67 antibody (Abcam, Cambridge, MA) overnight at 4°C. Slides were scanned with an Aperio Scanscope CS (Aperio Technologies, Inc., Vista, CA). Ki-67 positive cells in ten 20× fields were counted. For Lac-Z staining, liver cryosections (10 μm) were fixed in phosphate buffered saline, pH 7.3 (140 mM NaCl; 2.7 mM KCl; 8.1 mM Na2HPO_4_; 1.5 mM KH_2_PO_4_) containing 0.2% gluteraldehyde, 10 mM EGTA, and 2 mM MgCl_2_. Sections were washed three times with phosphate buffered saline containing 0.05 mM EGTA, 2 mM MgCl_2_, 0.12 mM Na deoxycolate, and 0.02% Nonidet P-40 and incubated overnight in the same solution containing 10 mM K_3_Fe(CN)_6_, 10 mM K_4_Fe(CN)_6_, and 0.5% X-gal. Sections were washed and counterstained with Nuclear Fast Red (Vector Labs; Burlingame, CA). Sections from *c-myc^+/+^;Alb*-Cre^-/-^;ROSA26^+ ^and *c-myc^+/+^;Alb*-Cre^+^;ROSA26^-/- ^mice served as negative controls.

### β-galactosidase analyses

β-gal ELISA assays were performed using a kit obtained from Roche (Indianapolis, IN). Briefly, livers were obtained from 6-8 week old *c-myc^fl/fl^*; *Alb*-Cre;ROSA26 mice and homogenized by hand using a ground glass homogenizer with 2 ml 1× Lysis buffer (Roche) per 40 mg tissue. Samples were incubated for 30 min at room temperature and centrifuged at 13,000 rpm for 15 min at 4°C. Samples were split into aliquots and frozen in dry ice/ethanol before transferring to -80°C. An aliquot was used to determine the protein concentration using the bicinchoninic acid method (Pierce; Rockford, IL). β-gal ELISA assays were performed according to the manufacturer's directions using 5 μg of liver homogenate.

### Statistical analyses

Statistical analyses were performed using GraphPad Prism (San Diego, CA). One-way ANOVA with a post hoc Bonferroni Multiple Comparison test was performed for comparing the relative abundance of the *c-myc^fl ^*allele by qPCR. Comparisons of the relative abundances of *c-myc *and *cre *expression in mice of various genotypes were performed using a Mann-Whitney test. A Spearman rank correlation test was performed to assess the association between *c-myc *and *cre *abundance. For results where differences were not significant, no *p *value is reported.

## Results

### Deletion of hepatic c-*myc*

Deletion of murine *c-myc *by gene targeting results in embryonic lethality at embryonic day 9-10 [23]. In order to assess the functional role of c-Myc in adult mouse liver, we utilized a transgenic mouse line where Cre recombinase is under the control of the *Albumin *promoter [18]. Conditional deletion of *c-myc *was achieved by crossing these mice with *c-myc^fl/fl ^*mice. The F1 generation was intercrossed to obtain *c-myc^fl/fl^;Alb*-Cre^+ ^and littermate control mice (*c-myc^fl/fl^;Alb*-Cre^-/-^, *c-myc^+/+^;Alb*-Cre^+^). The *c-myc^fl/fl^;Alb*-Cre^+ ^and *c-myc^+/+^;Alb*-Cre^+ ^lines were also bred to be homozygous for the Cre transgene. Litters contained the expected frequency of control and experimental animals.

**Figure 1:**
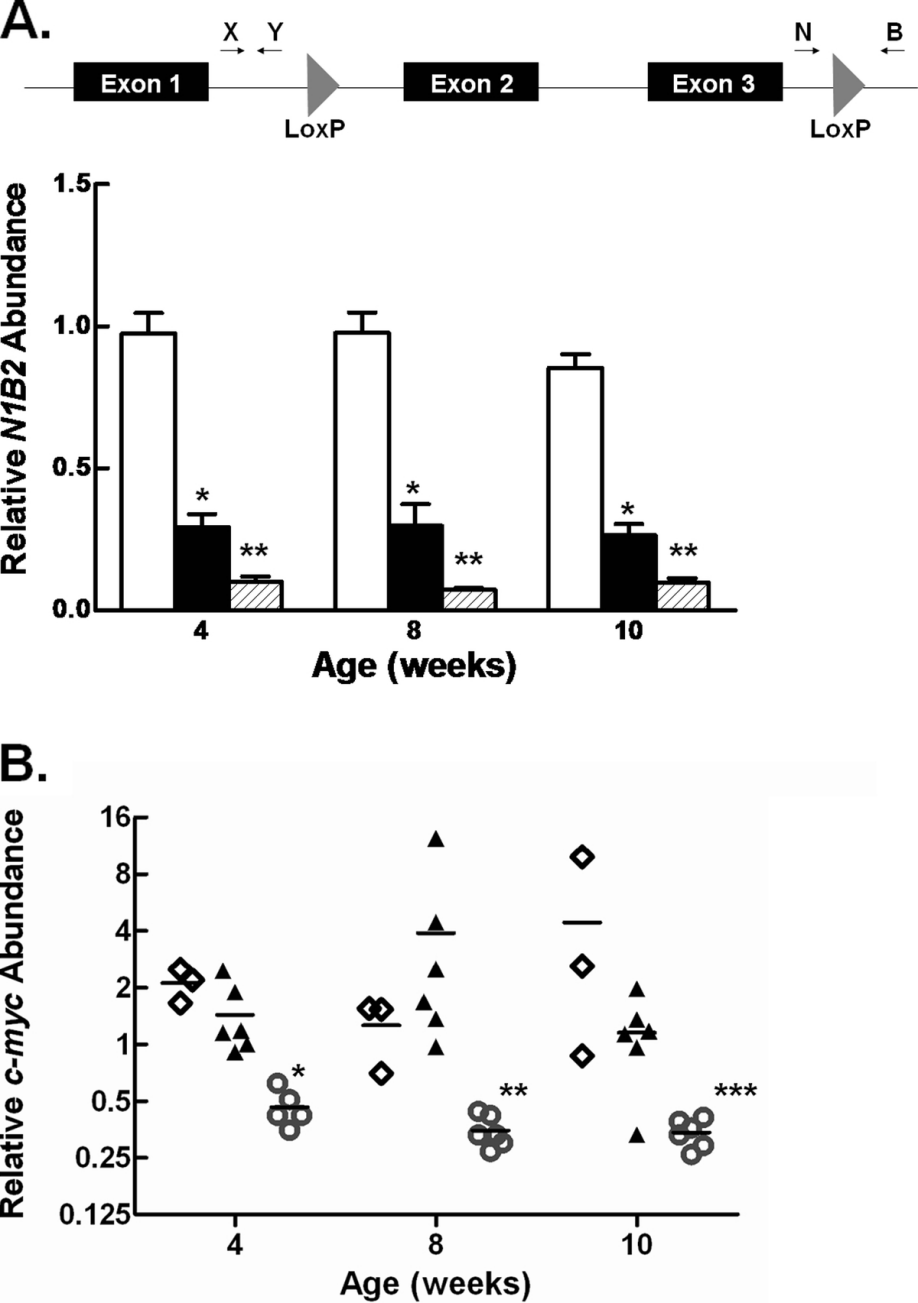
**Hepatic *c-myc *deletion in *c-myc^fl/fl^;Alb-Cre *expressing mice**. *A*. Schematic representation of the *c-myc^fl ^*allele showing location of primer pairs used in qPCR analyses to determine the relative abundance of the *c-myc^fl ^*allele. The graph shows the quantification of the *c-myc^fl ^*allele in liver from male and female *c-myc^fl/fl^;Alb-Cre^-/- ^*(open bars), *c-myc^fl/fl^;Alb-Cre^+ ^*(closed bars) and *c-myc^fl/fl^;Alb-Cre^+/+ ^*(hatched bars) mice as described in the Methods. Data are shown as the mean + 1 SD. *, P < 0.01 *c-myc^fl/fl^;Alb-Cre^+ ^*versus *c-myc^fl/fl^;Alb-Cre^-/- ^*. **, P < 0.001 *c-myc^fl/fl^;Alb-Cre^+ ^*compared to *c-myc^fl/fl^;Alb-Cre^+/+^*. *B*. Hepatic *c-myc *expression in male and female control and *c-myc^fl/fl^;Alb-Cre *expressing mice as analyzed by RT-qPCR. Data are shown as individual points with the mean. **◊**, *c-myc^fl/fl^;Alb-Cre^-/-^*; ▲, *c-myc^+/+^;Alb-Cre*; ○, *c-myc^fl/fl^;Alb-Cre*. *, P = 0.001 4 wk *c-myc^fl/fl^;Alb-Cre *vs. controls; **P = 0.0004 8 wk *c-myc^fl/fl^;Alb-Cre *vs. controls; ***P = 0.0028 10 wk *c-myc^fl/fl^;Alb-Cre *vs. controls.

Cre-mediated recombination of the *c-myc *locus was assessed using a qPCR strategy. Primers (XY) were designed to amplify a region of the *c-myc *locus upstream of the 5' loxP site. A second primer pair (NB) was designed surrounding the 3' loxP site such that Cre-mediated recombination of the locus would result in a decrease of the NB qPCR product (Figure 1A). Results revealed a significant decrease in the relative abundance of the NB product compared to the XY product in conditional knockout animals, indicating recombination of the *c-myc *locus.

By one month of age, recombination approached 70% in both male and female *c-myc^fl/fl^;Alb*-Cre^+ ^expressing mice, while recombination in *c-myc^fl/fl^;Alb*-Cre^+/+ ^mice was significantly higher (Figure 1A). Consistent with previous findings on the temporal expression of the *Albumin*-Cre transgene [24], a similar deletion efficiency was observed in 8 and 10 week old conditional knockout animals, indicating that maximal recombination had been reached by one month of age. Since hepatocytes make up approximately 85% of the total cell population in liver, we estimated that recombination of the *c-myc *locus was near 80% in *c-myc^fl/fl^;Alb*-Cre^+ ^and over 90% in *myc^fl/fl^;Alb*-Cre^+/+ ^mice.

To examine the influence of Cre-mediated recombination on *c-myc *mRNA, RT-qPCR was performed on total RNA isolated from livers obtained from control (*c-myc^+/+^;Alb*-Cre^+/+^, *c-myc^fl/fl^;Alb*-Cre^-/-^) and *c-myc^fl/fl^;Alb*-Cre^+/+ ^mice at 4, 8, and 10 weeks of age (Figure 1B). A 75% reduction in *c-myc *expression in livers from *c-myc^fl/fl^;Alb*-Cre^+/+ ^male and female mice was observed at one month of age and this reduction in c-*myc *expression remained stable in the liver through 10 weeks of age.

We assessed the effect of the model on c-Myc protein levels by immunoprecipitating c-Myc from total liver homogenates prepared from 4 week old *c-myc^fl/fl^;Alb*-Cre^+/+ ^and *c-myc^+/+^;Alb*-Cre^+/+ ^mice (data not shown). c-Myc protein content was low in four week old *c-myc^+/+^;Alb*-Cre^+/+ ^control mice and below the level of detection in *c-myc^fl/fl^;Alb*-Cre^+/+ ^mice.

### Characterization of hepatic c-myc knockout mice

**Figure 2:**
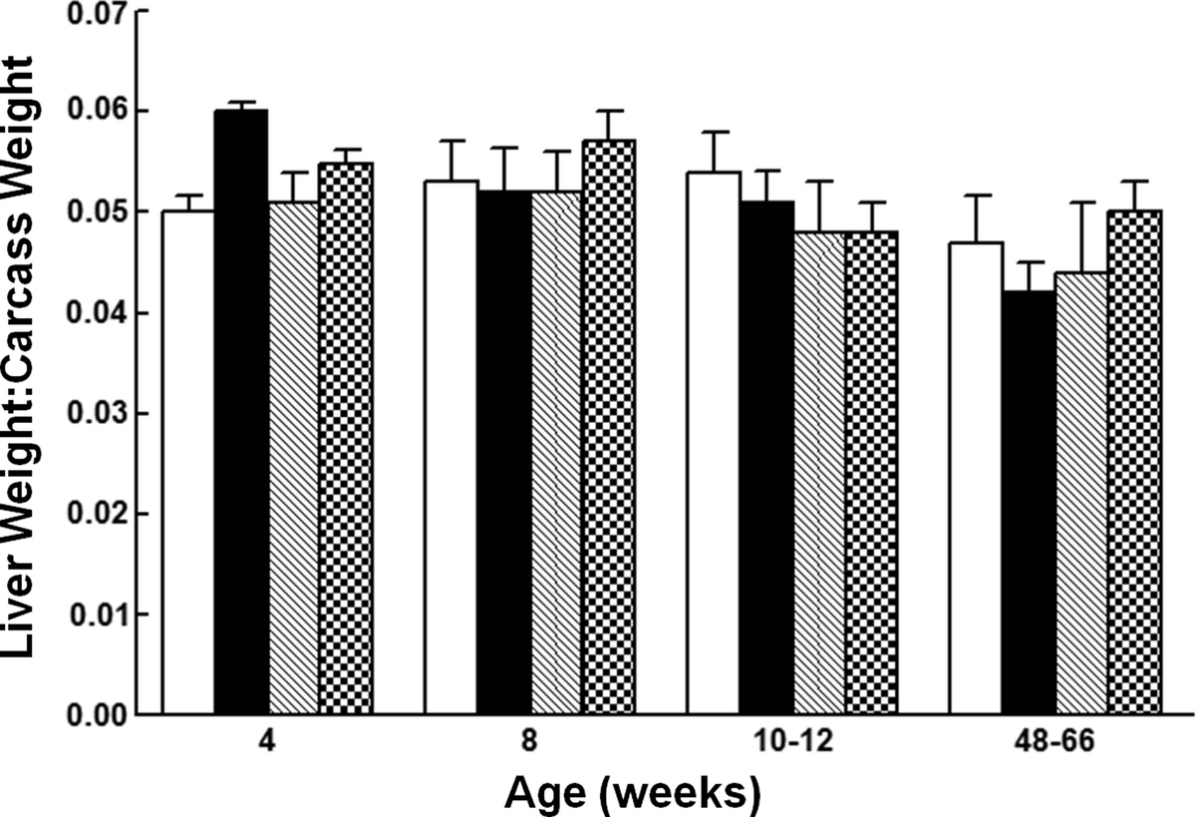
**Liver weight to carcass weight ratio in *c-myc^fl/fl^;Alb-Cre *expressing mice**. Liver:carcass weight ratio was determined for control and *c-myc^fl/fl^;Alb-cre *male and female mice at various ages. Data are expressed as mean + 1 SD. n ≥ 3 mice/group. Open bars, *c-myc^fl/fl^;Alb-Cre^-/-^*; Black bars, *c-myc^+/+^;Alb-Cre^+^*; Hatched bars, *c-myc^fl/fl^;Alb-Cre^+^*; Stippled bars, *c-myc^fl/fl^;Alb-Cre^+/+^*.

Our prior studies in the rat showed that c-Myc protein was expressed in quiescent adult hepatocytes, suggesting a functional role for the protein in adult liver other than its role in proliferation [16]. Given the established role of *c-myc *in hepatic proliferation and growth, we measured liver weight to carcass weight ratios in male and female *c-myc^fl/fl^;Alb-Cre^+^, c-myc^fl/fl^;Alb-Cre^+/+ ^*and control mice from one month of age through the first year of life. Liver weight to carcass weight ratios were similar in *c-myc^fl/fl ^Alb*-Cre expressing and control mice at all ages analyzed (Figure 2). Despite the increased recombination of the *c-myc *locus in *c-myc^fl/fl^;Alb-Cre *homozygous mice, no difference in liver weight was observed in these animals compared to *c- myc^fl/fl^;Alb-Cre *hemizygous mice.

c-Myc has been shown to regulate genes involved in glucose metabolism and to be involved in the regulation of cell and nucleolar size [12,25]. In order to determine if loss of c-Myc would result in alterations in glucose homeostasis we analyzed serum glucose levels in fed *c-myc^fl/fl ^*and *c-myc^+l+ ^Alb-Cre *expressing mice at 4 and 8 weeks of age. Serum glucose was unaffected in the *c-myc^fl/fl^;Alb-Cre *expressing mice at both ages (277 ± 19 vs. 271 ± 73.6 and 305 ± 27 vs. 295 ± 20; 4 and 8 weeks respectively).

To investigate whether the organization of the liver parenchyma or hepatocyte morphology was affected in *c-myc *conditional knockout mice, hematoxylin and eosin stained liver sections were prepared from *c-myc^fl/fl^;Alb-Cre *expressing and control mice at 4, 8, and 10 weeks of age (data not shown). The gross and histological appearance of the liver were similar in *c-myc^fl/fl^;Alb-Cre *expressing and control animals as well as *c-myc^fl/fl^;Alb-cre *hemizygous compared to homozygous animals.

### c-*myc^fl/fl^;Alb-Cre *expressing hepatocyte response to nonproliferative and proliferative stimuli

**Figure 3:**
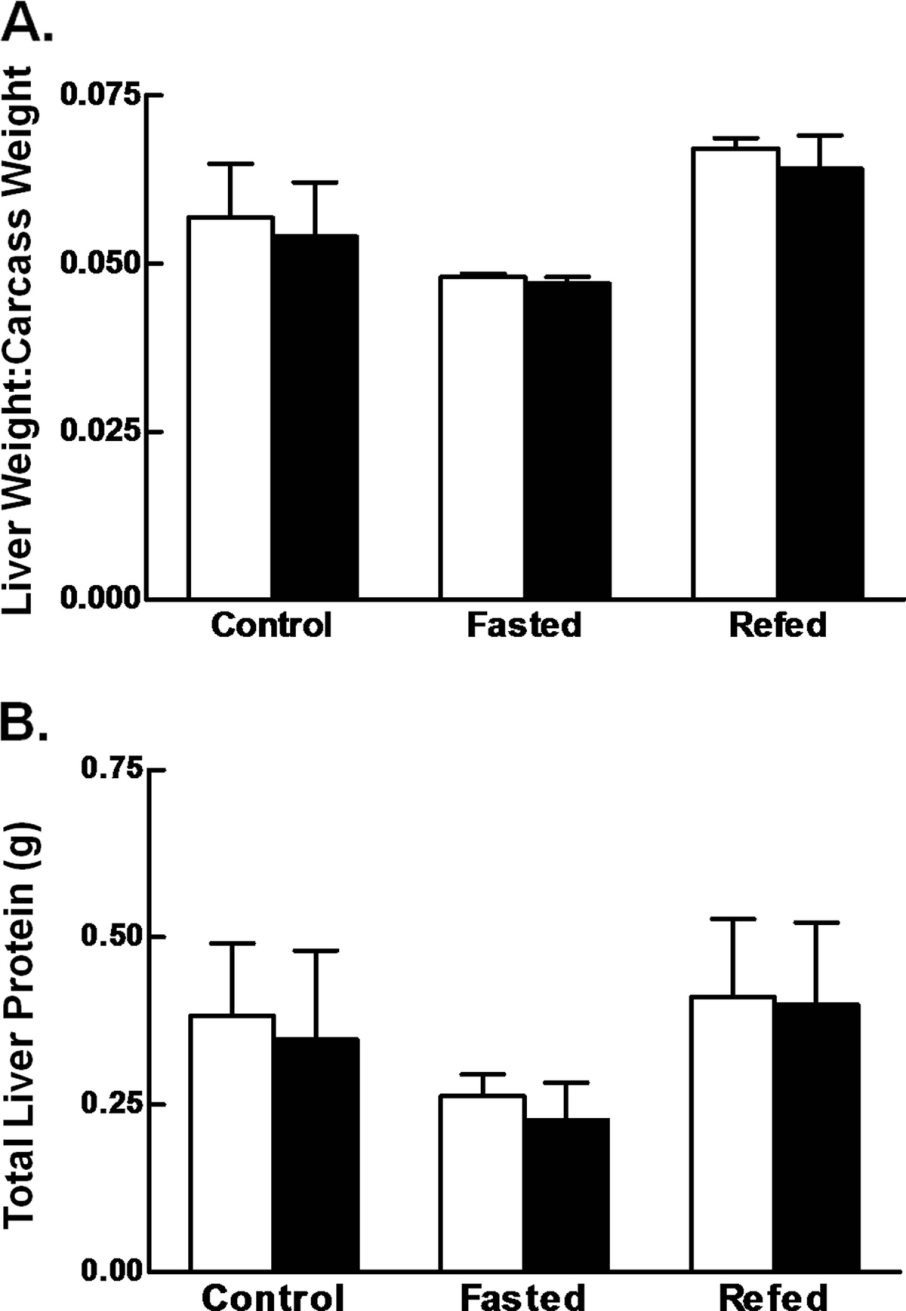
**Effect of conditional *c-myc *deletion on liver growth following starvation**. Male and female mice were fed ad libitum (control), fasted for 48 hr (fasted), or fasted for 48 hr then refed ad libitum for 24 hr (refed). A. The graph shows liver:carcass weight ratios for *c-myc^+/+ ^*(open bars) and *c-myc^fl/fl ^*(closed bars) *Alb-Cre *expressing mice expressed as the mean + 1 SD. n ≥ 3 mice/group. B. Liver homogenates were prepared and protein concentration determined using the bichoninic acid method. Total liver protein was then calculated based on total liver weight. The graph shows total liver protein content for *c-myc^+/+ ^*(open bars) and *c-myc^fl/fl ^*(closed bars) *Alb-Cre *expressing mice expressed as the mean + 1 SD. n ≥ 3 mice/group.

We extended our studies to determine the effect of reducing c-Myc in a nonproliferative model of liver growth, refeeding after a 48 hr fast. Previous studies in our laboratory have shown that rat liver growth during refeeding requires rapid and marked induction of ribosomal biogenesis and translation initiation, two processes where c-Myc has been proposed to have a functional role [26]. Eight week old *c-myc^fl/fl ^*and *c-myc^+l+ ^Alb*-Cre expressing mice of both sexes were divided into three groups. Mice were either fed *ad libitum *(control), fasted for 48 hr, or refed *ad libitum *for 24 hr following a 48 hr fast (refed). As no difference in liver weight to carcass weight ratio or total liver protein was observed in *c-myc^fl/fl ^*animals carrying one or two alleles of the *Alb-Cre *transgene, the data from these animals were combined. In agreement with our previous data in the rat [26], fasting for 48 hr resulted in a slight reduction in liver to carcass weight ratio in both control and *c-myc^fl/fl ^Alb-*Cre expressing animals. Refeeding for 24 hr resulted in restoration of liver weight to carcass weight ratio to the level of the control fed mice in both *c-myc^fl/fl ^*and *c-myc^+l+ ^*animals (Figure 3A). Total liver protein decreased by approximately 40% in both genotypes with fasting. Recovery of total protein was attained after 24 hr of refeeding in both genotypes, suggesting that full expression of *c-myc *is not required for protein synthesis in this model of liver growth (Figure 3B).

**Figure 4:**
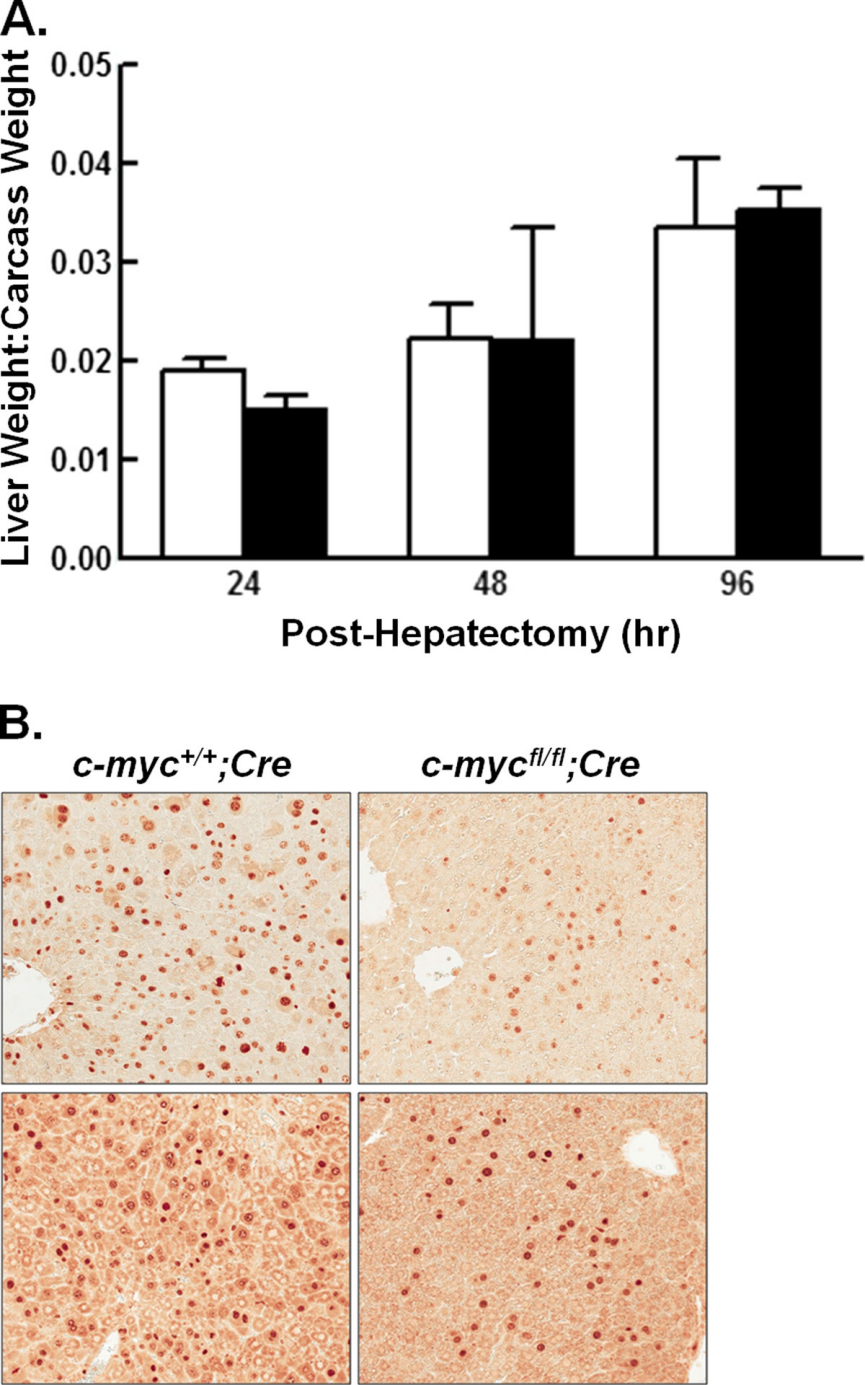
**Effect of conditional *c-myc *deletion on liver regeneration**. Male mice were subjected to 2/3 hepatectomy and assessed 24, 48, or 96 hr after resection. A. The graph shows liver:carcass weight ratio for *c-myc^+/+ ^*(open bars) and *c-myc^fl/fl ^*(closed bars) *Alb-Cre *expressing mice. The data are expressed as the mean + 1 SD. n ≥ 3 mice/group. B. Hepatocyte proliferation in duplicate *c-myc^+/+ ^*and *c-myc^fl/fl ^Alb-Cre *expressing mice 48 hr post-hepatectomy as assessed by Ki-67 immunohistochemistry. Representative 20× images are shown.

**Figure 5:**
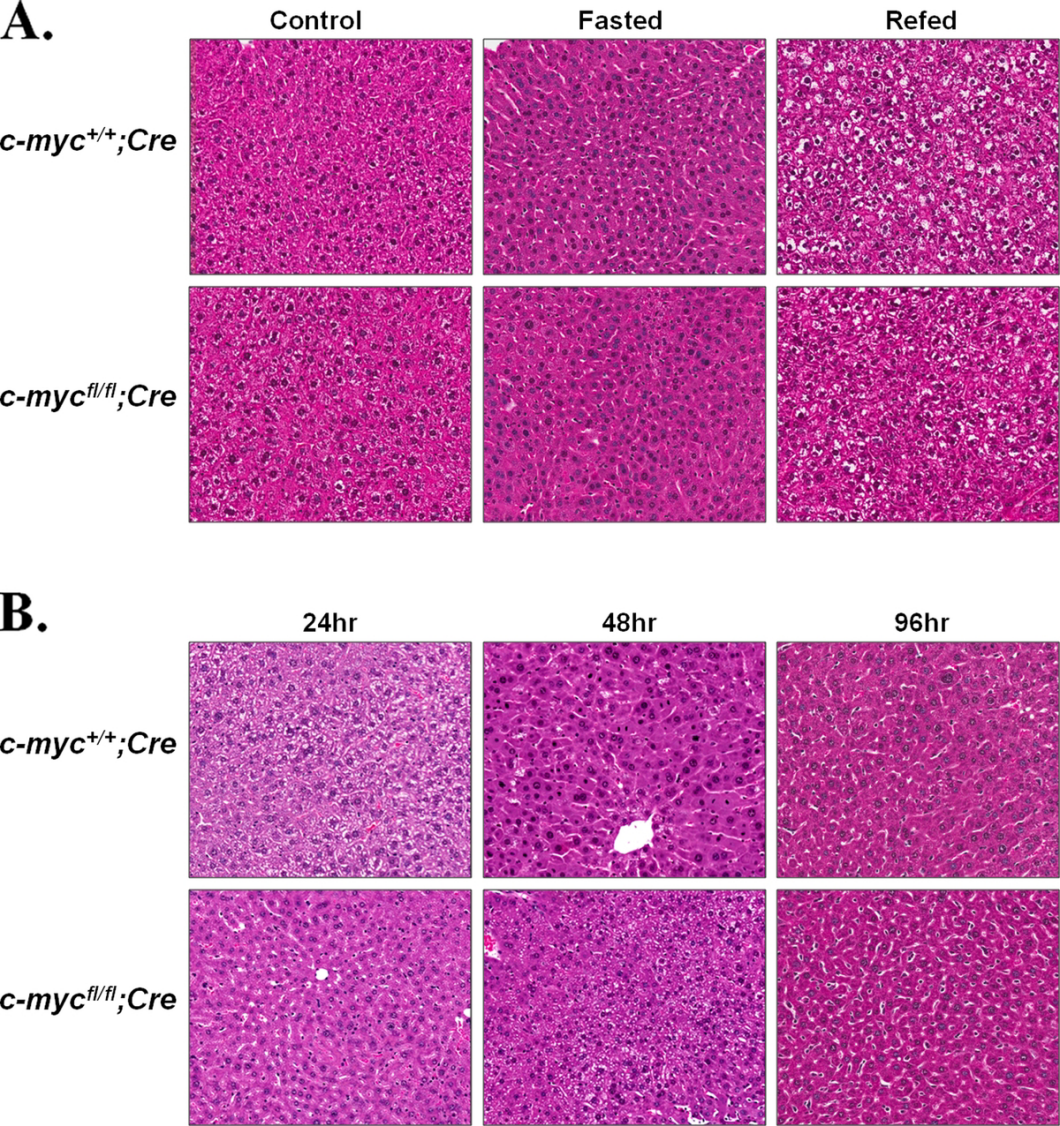
**Effect of conditional *c-myc *deletion on parenchymal organization during fasting/refeeding and liver regeneration**. Formalin fixed liver from male and female mice was processed and stained with H&E. Slides were scanned with an Aperio Scanscope CS and randomly selected representative areas with a scan zoom of 20 were acquired. A. Images (20×) obtained from control, 48 hr fasted, and 48 hr fasted followed by 24 hr refeeding *c-myc^+/+ ^*and *c-myc^fl/fl ^Alb-Cre *expressing mice. B. Representative 20× images from *c-myc^+/+ ^*and *c-myc^fl/fl ^Alb-Cre *expressing mice 24, 48, or 96 hr post-hepatectomy.

Quiescent adult hepatocytes rapidly enter the cell cycle in response to a reduction in liver mass. Upregulation of c-Myc protein content is an early event in this process, suggesting the hypothesis that liver regeneration following partial hepatectomy requires c-Myc [11,15]. To test this hypothesis, eight to ten-week old male *c-myc^+l+ ^*and *c-myc^fl/fl ^Alb-Cre *expressing animals underwent 2/3 partial hepatectomy. Mice were sacrificed 24, 48, or 96 hr post-hepatectomy. Liver weight to carcass weight ratios were determined (Figure 4A). No difference in liver to carcass weight ratio between *c-myc^fl/fl^;Alb-Cre *expressing and control mice was observed at any of the timepoints analyzed. However, a slight decrease in the number of Ki-67 positive hepatocytes was observed in the *c*-*myc^fl/fl ^*mice compared to wild-type mice 48 hr post-hepatectomy (Figure 4B). To determine if cells lacking in *c-myc *were responsible for liver regeneration, we quantified *c-myc *deletion in each mouse in the regenerated liver and liver excised at the time of partial hepatectomy. We found that the regenerating liver was as deficient in the *c-myc *allele as was the liver excised at the time of surgery [average fold change (0.84 ± 0.82; 0.80 ± 0.93; 0.78 ± 0.86) 24, 48, 96 hr, respectively]. The persistent loss of the *c-myc *allele was consistent with the conclusion that the recovered liver mass was arising from *c-myc*-deficient cells. Despite any modest decrease in the number of hepatocytes in the cell cycle, the absence of an effect on the recovery of liver weight indicates that the liver is capable of regeneration despite a significant reduction in *c-myc*. As in the non-proliferative model, there was no difference in mice containing one versus two alleles of the *Alb-Cre *transgene. Liver sections obtained from mice placed on the fasting/refeeding protocol and from animals subjected to 2/3 partial hepatectomy did not reveal disorganization of the liver parenchyma or any other obvious changes in hepatocyte morphology between *c-myc^fl/fl ^*and *c-myc^+l+ ^Alb-Cre *expressing mice (Figure 5).

### Expression of members of the *c-myc/max/mad *network

**Figure 6:**
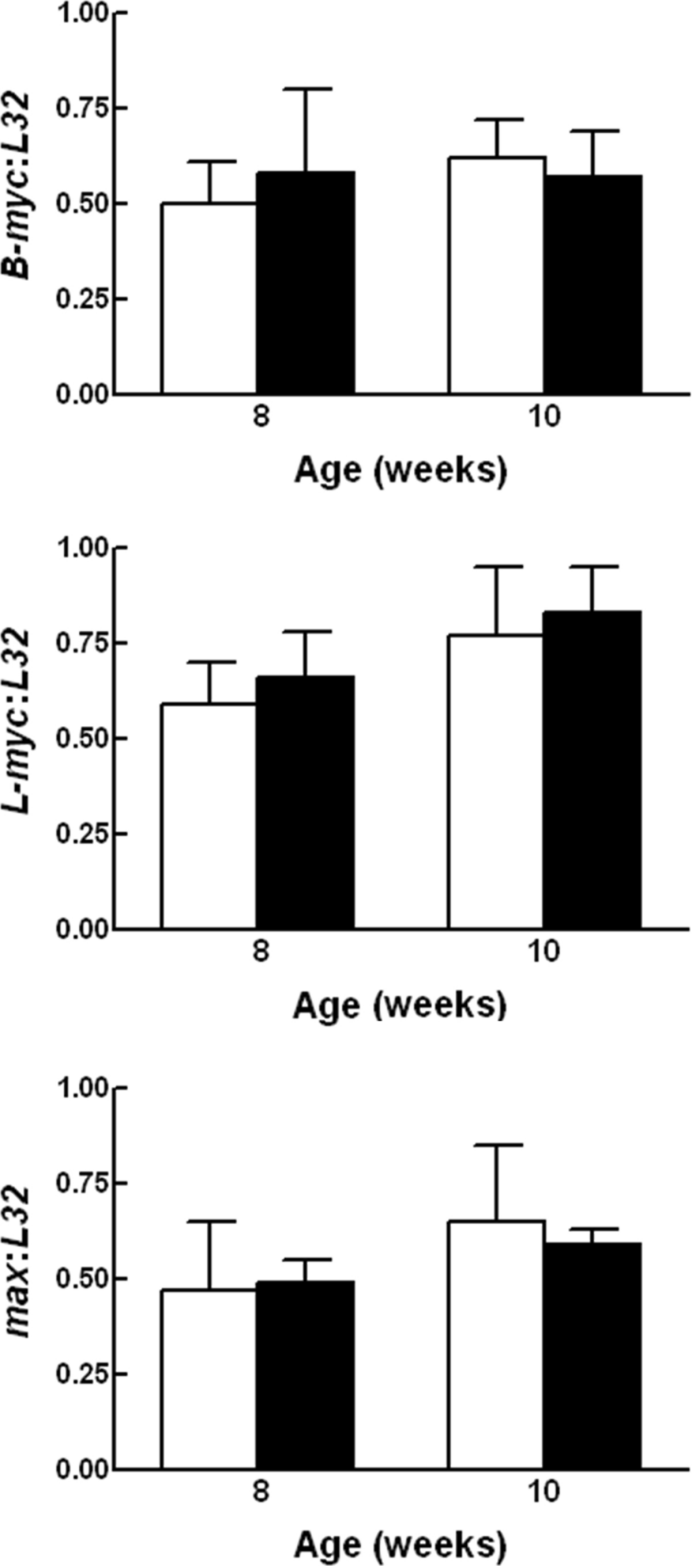
**Expression of members of the *c-myc*/*max*/*mad *network in liver derived from male and female control and *c-myc *conditional knockout mice**. Total liver RNA was isolated from triplicate *c-myc *wild-type (open bars) and *c-myc^fl/fl ^*(closed bars) *Alb-Cre *expressing mice at 8 and 10 weeks of age and a multiprobe RNase protection assay performed. Expression levels of *B-myc, L-myc*, and *max *were quantified and normalized to the internal control *L32*. Data are expressed as mean + 1 SD.

We went on to explore whether the lack of an effect of reducing *c-myc *on hepatocyte proliferation, growth, and protein synthesis could be accounted for by compensatory induction of other *myc *family members or the c-Myc binding partner Max. We had previously found that *max *expression at both the RNA and protein level correlated with hepatocyte proliferation during rat liver development and that overexpression of *max *induced a shift in c-Myc localization from the nucleolus to the nucleus [15]. These data raise the possibility that compensatory induction of Max could increase c-Myc activity. Multiplex RNase protection assays were performed on total RNA isolated from 8 and 10 week old *c-myc^fl/fl ^*and *c-myc^+l+ ^Alb-*Cre expressing animals of both sexes (Figure 6). Two of the *myc *family members, *B-myc *and *L-myc *were expressed in murine liver, while *N-myc *expression was below the level of detection in our assay. There was no difference in the expression of these family members or of *max *in *c-myc^fl/fl ^*compared to wild-type *c-myc *Alb-Cre expressing mice.

There is substantial overlap between the role of c-Myc and Wnt/β-catenin signaling in the regulation of postnatal liver development, hepatic organization, and liver regeneration [27]. In addition, c-myc is a downstream target of β-catenin [28]. Given the interaction between c-myc and β-catenin, we investigated whether deletion of *c-myc *is compensated by upregulation of β-catenin signaling. Immunohistochemical analysis for β-catenin was performed on liver sections from wild-type and *c-myc *deficient mice. No difference in the localization of β-catenin was observed between the two genotypes of mice. These results were confirmed by Western immunoblotting of nuclear and post-nuclear fractions prepared from liver of wild-type and *c-myc *deficient mice (data not shown).

### Effect of *c-myc^fl/fl ^*on Cre expression

**Figure 7:**
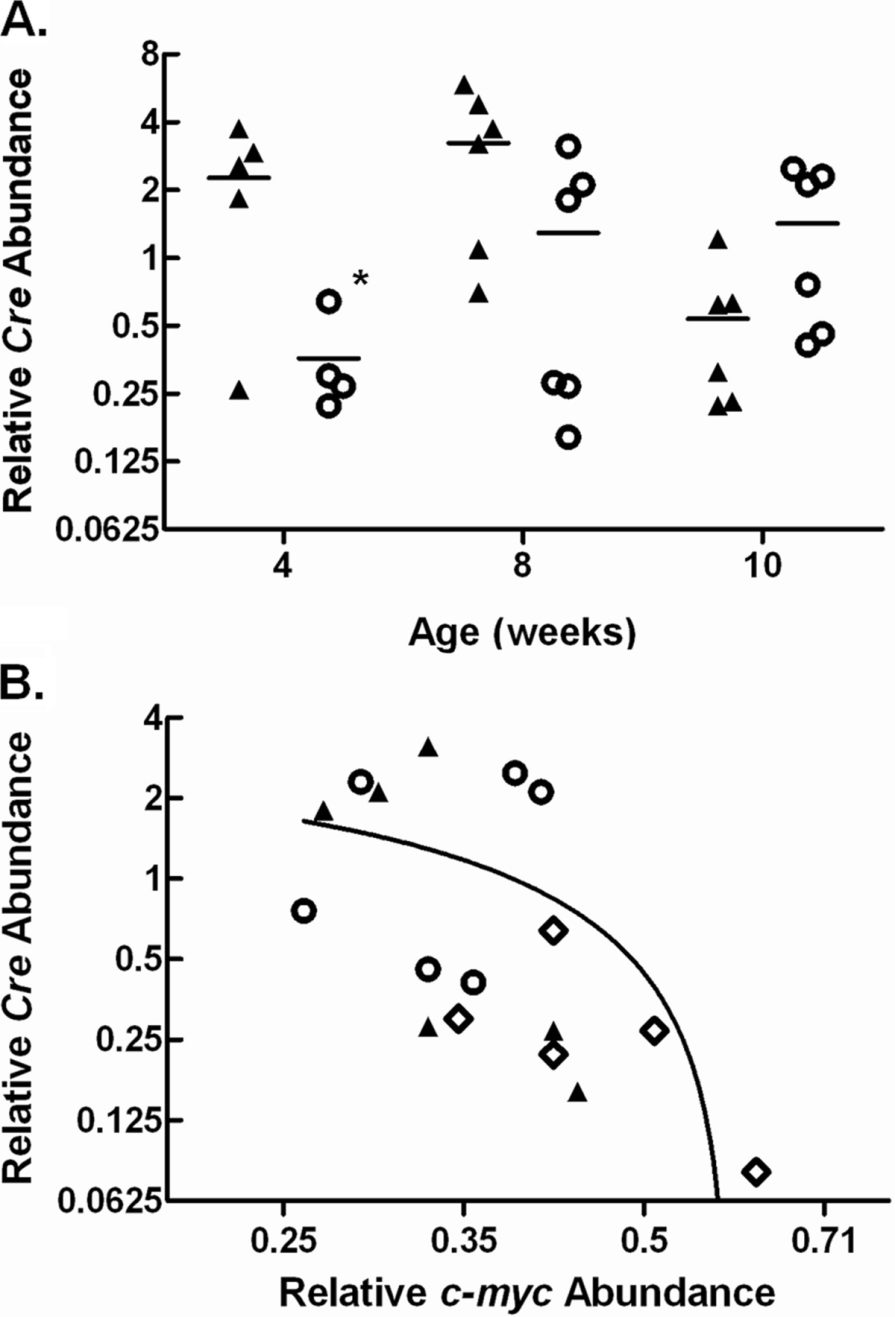
**Cre expression in *c-myc *conditional knockout and control mice**. A. Total liver RNA was isolated from male and female *c-myc^+/+^;Alb-Cre^+/+ ^*(▲) *and c-myc^fl/fl^;Alb-Cre^+/+ ^*(○) mice and *cre *expression assessed using RT-qPCR. Data are shown as individual points with the mean. *, P = 0.0303 4 wk *c-myc^fl/fl^;Alb-Cre^+/+ ^*vs control. B. The correlation between *cre *expression and *c-myc *abundance in *c-myc^fl/fl^;Alb-Cre^+/+ ^*expressing mice at 4, 8 and 10 weeks of age. Data are expressed as individual points. **◊**, 4 weeks; ▲, 8 weeks; ○, 10 weeks.

Numerous studies using the *Alb*-Cre transgene to delete various genetic loci involved in hepatocyte proliferation, growth, and survival have reported recombination nearing 100% and complete ablation of the expression of the gene of interest [29-31]. Given our results, which varied markedly from published experience, we investigated the expression and activity of the *Alb*-Cre transgene in *c-myc^+/+ ^*and *c-myc^fl/fl ^*mice to ascertain whether floxing *c-myc *had an effect on the expression of the *Alb*-Cre transgene such that *c-myc *deletion would be impaired in our model. RNA was isolated from livers obtained from *c-myc^+/+^;Alb-Cre^+/+ ^*and *c-myc^fl/fl^;Alb-Cre^+/+ ^*animals at 4, 8, and 10 weeks of age. RT-qPCR was performed to assess *cre *expression (Figure 7A). A statistically significant difference in *cre *expression was observed in *c-myc^fl/fl ^*compared to *c-myc^+/+ ^*mice at 4 weeks of age suggesting that deletion of *c-myc *suppressed expression of the *Alb-Cre *transgene. At 8 and 10 weeks of age there was no statistically significant difference in *cre *expression between *c-myc^fl/fl ^*and *c-myc^+/+ ^*mice. However, in comparison to 4-week old animals, *cre *expression in older mice varied considerably from animal-to-animal.

To ascertain whether the variation in *cre *expression correlated with the amount of c-*myc *we compared the relative abundance of *cre *and *c-myc *in individual *c-myc^fl/fl^;cre^+/+ ^*mice at 4, 8, and 10 weeks (Figure 7B). This analysis revealed a significant association between floxing *c-myc *and the expression of *cre *(Spearman r = -0.6609, p = 0.0019).

**Figure 8:**
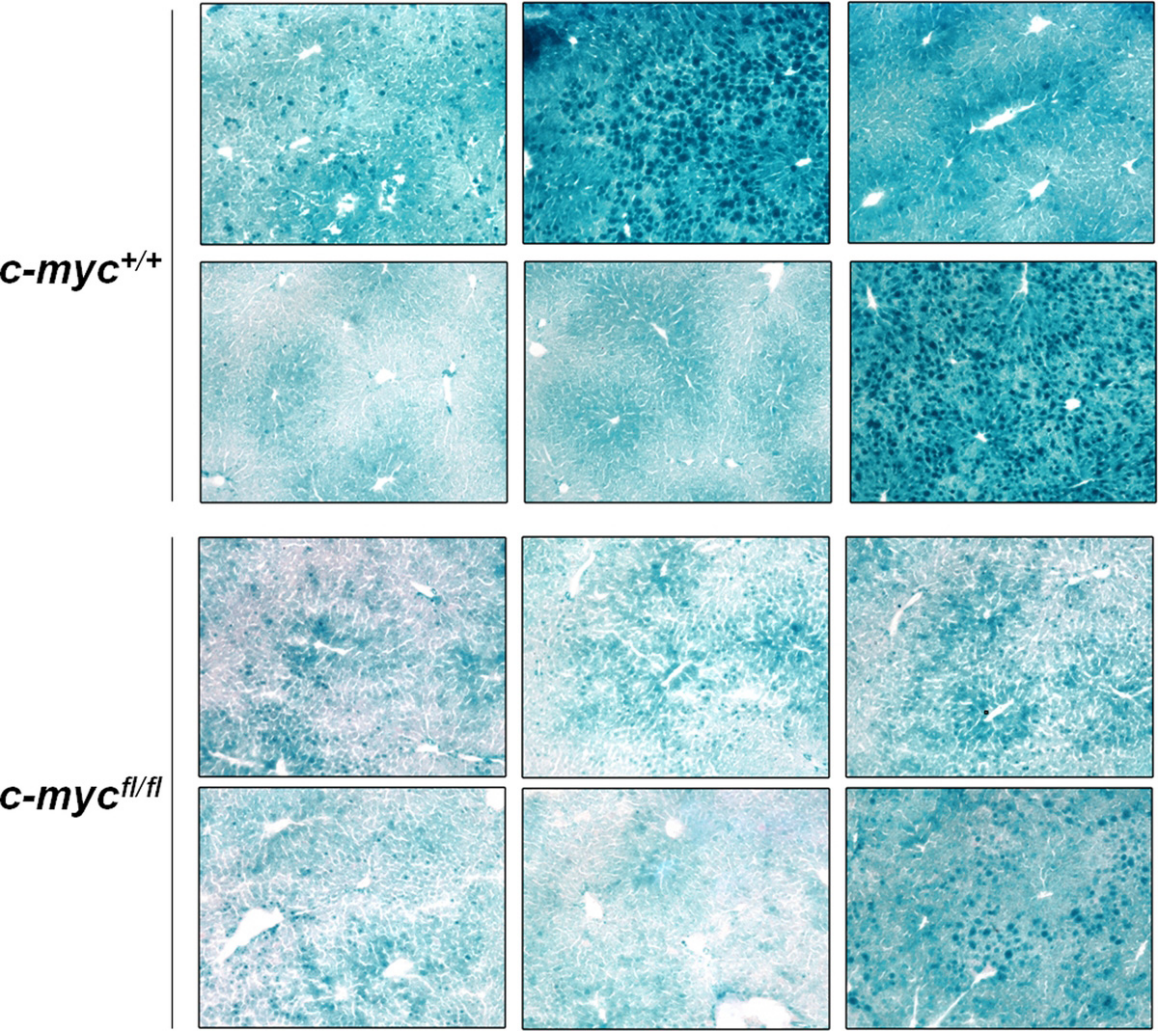
**Cre activity in *c-myc *conditional knockout and control mice**. Cre expressing c-*myc^+/+ ^*and *c-myc^fl/fl ^*mice were crossed to mice carrying the Rosa26R allele to obtain *c-myc^+/+^;Rosa*;*Alb-Cre *and *c-myc^fl/fl^;Rosa*;*Alb-Cre *mice. Liver cyrosections (10 μm) from male and female mice were fixed and stained for Lac-Z. Photomicrographs of three 20× fields from individual sections acquired from *c-myc^+/+^;Rosa*;*Alb-Cre *and *c-myc^fl/fl^;Rosa*;*Alb-Cre *mice are shown. All panels show images that are representative of the results obtained on multiple sections from at least 2 animals per group. The results were replicated in a duplicate experiment. The contrast was adjusted across the entire image to accurately reflect the appearance of the sections under the microscope.

We further characterized the pattern of Cre activity in our model by crossing our mice to the ROSA26 reporter line. The ROSA26 line has a lacZ gene that is expressed when an upstream stop codon is removed by Cre-mediated recombination. In order to study the effect of floxing *c-myc *on Cre activity, mice were further crossed to obtain *c-myc^fl/fl^;Alb*-Cre;ROSA26 and *c-myc^+/+^;Alb*-Cre;ROSA26 mice. Sections prepared from six-week old mice were stained for β-galactosidase activity using X-gal. Analysis revealed a wide pattern of LacZ-staining across 20× fields of individual sections in both genotypes of mice (Figure 8). In some fields, the majority of the hepatocytes displayed intense nuclear and cytoplasmic staining while other hepatocytes exhibited weak cytoplasmic staining. There were also fields where many of the hepatocytes appeared negative for Lac-Z staining. In light of the qualitative nature of the Lac-Z staining, we prepared liver homogenates from 6-8 week old mice and performed β-gal ELISA assays in order to quantitate Cre activity (data not shown). No difference in B-gal content was found in the *c-myc^fl/fl^;Alb*-Cre;ROSA26 versus the c-*myc^+/+^;Alb*-Cre;ROSA26 controls.

## Discussion

The transcription factor c-Myc has long been assigned a prominent role in the synchronous hepatocyte proliferation that occurs during liver regeneration [9,11]. A series of *in vivo *studies performed in our laboratory characterizing the regulation of the *c-myc/max*/*mad *network in fetal and adult liver revealed that c-Myc was present in quiescent adult hepatocytes and was localized to the nucleolus [16]. These studies led us to hypothesize a functional role for c-Myc in adult liver that was independent of its role in proliferation. In order to test this hypothesis, we generated a conditional knockout using floxed *c-myc *and *Albumin*-*Cre *mice. This approach led to significant reduction in *c-myc *expression one month after birth in *c-myc^fl/fl^;Alb*-Cre expressing mice. We observed greater recombination efficiency in *c-myc^fl/fl^;Alb-Cre *homozygous compared to hemizygous animals. However, there was no difference in liver weight ratios during development in *c-myc^fl/fl^;Alb-Cre*^+/+ ^compared to *c-myc^fl/fl^;Alb-Cre*^+ ^animals. As we did not observe differences in these two groups in regards to histology, liver regeneration or the recovery from fasting, the animals were grouped together for comparison with *c-myc *wild-type *Alb-Cre *expressing mice. There was a low level of residual *c-myc *that persisted in *c-myc^fl/fl^;Alb*-*Cre *expressing livers even up to four months. This residual level may be a result of expression in nonparenchymal cells or a subset of hepatocytes in which the albumin promoter is not expressed. However, we were unable to exclude a low level of *c-myc *expression in the larger population of hepatocytes. Interestingly, floxed *c-myc *had an inhibitory effect on *Cre *expression in one month old mice. In contrast, in older animals *Cre *expression was extremely variable regardless of *c-myc *status. This variation may be a result of age-dependent silencing of the *Cre *transgene.

Variegation of transgene expression is a well documented phenomenon in many lines of transgenic mice [32-34] although the cause and mechanism is not known. In a mouse model where *lacZ *was driven by the *β-globin *promoter, there was a general tendency of decreased transgene expression with age [35]. In contrast to other reports on variable transgene expression, we observed a negative effect of our floxed gene on *Cre *recombinase expression. We speculate that this effect is a result of a selective pressure to retain *c-myc *in hepatocytes. It is possible that this selective effect could manifest itself in ways other than an effect on Cre expression.

There was no apparent phenotypic effect of significantly reducing *c-myc *in hepatocytes. The livers of *c-myc^fl/fl^;Alb*-*Cre *expressing and control mice were of similar size and histology, consistent with the conclusion that *c-myc *is not required for hepatocyte proliferation during normal liver growth and maintenance. We used two models, partial hepatectomy and liver growth following refeeding to determine c-Myc function in the presence or absence of a proliferative stimulus. c-Myc has been considered to have a prominent role in the hepatocyte proliferation that occurs during liver regeneration. After partial hepatectomy there was a slight decrease in the number of hepatocytes in the cell cycle at 48 hr. However, no delay in the restoration of liver mass was observed, indicating that *c-myc *is not required for liver regeneration following 2/3 partial hepatectomy. While these studies were in progress, two conflicting reports were published on the effect of deleting *c-myc *on liver regeneration. Baena et al. found that deletion of *c-myc *resulted in impaired liver regeneration [36]. However, Li et al. reported a total restoration of liver mass by 7 days post-resection in mice where floxed *c-myc *was deleted using adenoviral Cre [37]. In contrast to our study and Li et al., where liver/body mass ratio was used as the outcome measurement for liver regeneration, Baena et al. used PCNA and Cyclin A content as an indirect measure of hepatocyte proliferation. Furthermore, the content of these proteins was only determined 2 day post-hepatectomy leaving it unclear whether liver regeneration would have been affected at later time points. Taken together, these studies indicate that *c-myc *is not essential for restoration of liver mass during regeneration.

In some systems, changes in *c-myc *content affect cell size without affecting cell proliferation [38]. In order to assess the function of c-Myc in a non-proliferative model of hepatocyte growth, mice were fasted for 48 hr followed by a 24 hr refeeding period. In accordance with our published data in the rat, a 48 hr fast resulted in decreased liver/body mass ratio and liver protein content while refeeding resulted in restoration of liver mass and protein despite a significant reduction in *c-myc*. c-Myc has been proposed to play a role in essential processes leading to hepatocyte growth, such as, ribosomal biogenesis and protein synthesis. Kim et al found that transient *c-myc *overexpression in mouse liver led to hepatocyte hypertrophy, the induction of ribosomal genes, and increased protein synthesis [12]. Our results indicate that protein synthesis and hepatocyte growth can occur in spite of a significant reduction in *c-myc*, raising the notion that overexpression of c-Myc may result in the activation of gene regulatory networks and pathways not normally controlled by c-Myc in adult hepatocytes.

Studies by Murphy et al. led to the conclusion that the level of *c-myc *expressed in a cell is crucial to its biological effect [39]. These studies used a mouse model where the activation of Cre recombinase results in the expression of a tamoxifen inducible MycER fusion protein with the amount of c-Myc expressed dependent on whether the mouse carried one or two MycER alleles. The authors report that modest increases in c-Myc can activate cellular proliferation while a higher threshold is needed to stimulate apoptosis. This study suggests that a high level of c-Myc may lead to the binding of a different set of target genes from those regulated by endogenous levels of c-Myc. This notion is further supported by studies on the role of c-Myc in hepatocarcinogenesis. Deregulation of c-Myc through gains in copy number, point mutations, and transactivation by viral proteins is observed in 30-60% of human hepatocellular carcinomas (HCC) [40,41]. Although its role in the development of human HCC is unclear, studies in transgenic mice have shown that overexpression of this oncogene results in increased hepatocyte proliferation, genomic instability and apoptosis. The paradoxical activation of cellular proliferation and growth in concert with apoptosis leads to the requirement of secondary mutations for tumor development [42,43].

Our studies do not rule out a subtle effect of deleting *c-myc *on other aspects of liver physiology. It is possible that *c-myc *deletion affects other pathways. c-Myc has been shown to regulate many genes involved in liver metabolism and can ameliorate the effects of diabetes on glucose metabolism in mice [25,44]. Regardless of potential effects on other pathways overall adult liver physiology appeared to be unaffected by *c-myc *deletion.

Studies performed to assess the requirement and function of c-Myc in other mature tissues suggest that the role of this protein in proliferation, growth, and other cellular processes is cell-type dependent. Deletion of *c-myc *in the hematopoietic lineage results in defective hematopoiesis and angiogenesis leading to embryonic lethality while there was no requirement for *c-myc *in endothelial cells [45]. Moreover, normal adult intestinal homeostasis occurs in the absence of *c-myc*, yet *c-myc *is required for the formation of intestinal crypts [46]. These studies lead to the conclusion that the *in vivo *targets of *c-myc *will vary based on cell type and developmental stage thus adding another layer of complexity to understanding the functional role of *c-myc*.

## Conclusions

Our studies indicate that a reduction in hepatic *c-myc *does not affect normal postnatal liver growth and development. Furthermore, reducing this proto-oncogene does not affect the restoration of liver mass during liver regeneration or the restoration of liver protein following fasting. However, our studies do not rule out subtle effects of *c-myc *on liver metabolism or liver physiology. Decreasing *c-myc *correlated with a reduction in the expression of the *Albumin-Cre *transgene, suggesting a selective pressure to maintain *c-myc*. Furthermore, this pressure may prevent the complete deletion of hepatic *c-myc *by traditional conditional knockout strategies.

## Competing interests

The authors declare that they have no competing interests.

## Authors' contributions

JAS, JMS and PG conceived the study. JAS performed all practical aspects of the study. CS contributed to design and analysis of qPCR assays. The manuscript was prepared by JAS and PG. All authors read and approved the final manuscript.
